# Student nurse perceptions of family nursing practices in South Africa: A descriptive survey

**DOI:** 10.4102/hsag.v29i0.2321

**Published:** 2024-03-28

**Authors:** Geldine Chironda, Petra Brysiewicz

**Affiliations:** 1 School of Nursing and Public Health, College of Health Sciences, University of KwaZulu–Natal, Durban, South Africa

**Keywords:** nursing perceptions, family nursing, family-nursing relationships, student nurses, South Africa

## Abstract

**Background:**

Family nursing practices (FNPs) are gaining momentum in global literature, but the available research has targeted qualified nursing professionals. There are limited studies exploring this phenomenon in undergraduate student nurses in South Africa.

**Aim:**

The study aimed at exploring the undergraduate student nurse perceptions of FNPs.

**Setting:**

The study was conducted at a selected university in KwaZulu-Natal, South Africa.

**Methods:**

A descriptive survey design was used to purposively select undergraduate nursing students. The Family Nursing Practice Scale (FNPS) was used to collect data online. Descriptive and inferential statistics were used to analyse quantitative data. Open ended questions were analysed using content analysis.

**Results:**

Out of 154 participants, 77 responded to the questionnaire, translating to a response rate of 50%. Compared with other studies in literature, student nurses rated their overall FNP as being low (M = 3.43, s.d. = 0.99). A further descriptive analysis revealed better FNPs (2.97) for 3rd year compared to 2nd year (3.90) nursing students with significance differences in the means (*p* < 0.0001). While family conflict, maintaining confidentiality, ill prepared and absent family were challenges experienced in FNP, advantages included obtaining detailed information, ability to plan individualised care and enhanced student nurse-family relationship.

**Conclusion:**

A lower critical practice appraisal and lower perceptions of interaction and reciprocity in the nurse-family relationship were identified. There is need for an inclusive curriculum that promotes and advocates for family nursing within the undergraduate programme.

**Contribution:**

This study highlights the importance of teaching family nursing to undergraduate student nurses.

## Introduction

Families play a major role in healthcare systems, and it is thus important for nurses to not only focus on the patient but also engage with their respective families (Chironda & Brysiewicz 2021; Hagedoorn et al. 2021). Family involvement in healthcare services has been shown to be an essential component of nursing care, which benefit both the family and patient (Svavarsdottir et al. 2018). The growing body of available literature globally reveals fewer adverse events, better self-management, fewer diagnostic tests, decreased use of health services and shorter lengths of stay in the hospital to be associated with family engagement in care (Aronson et al. 2009; Abrams et al. 2013; Goodridge et al. 2018). In addition, the quality of nurse-patient-family relationships, overall improvement in health literacy, self-care in chronic disease management, clinical decision-making and patient safety are also enhanced (Svavarsdottir et al. 2018).

Nursing professionals, including student nurses, therefore need to identify and meet healthcare needs of families to promote family engagement – a fundamental goal of family nursing (Imanipour & Kiwanuka 2020). Hence, the concept of family nursing practice (FNP) becomes a central goal in facilitating family nurse relationships, consequently family engagement in healthcare settings (Misto 2018). Nurses, including the student nurses they preceptor during clinical placements, are expected to reflect on their attitudes in working with families including critically appraising their family nursing practices in terms of knowledge, skill, confidence and satisfaction. Similarly, promoting interaction and reciprocity in the nurse-family relationship (NFR) in terms of planning care, promoting family participation and recognising biases and reciprocity in the therapeutic relationship is also crucial (Misto 2018; Simpson & Tarrant 2006).

While much of the current emphasis of nursing care practice is on families (Broekema et al. 2021; Eggenberger & Sanders 2016; Imanipour & Kiwanuka 2020; Meiers, Eggenberger & Krumwiede 2018), it remains a challenge for student nurses to involve families in care (Fitzgerald & Ward 2019). This is magnified in the African countries where family nursing is not a recognised specialty area of nursing (Chironda, Jarvis & Brysiewicz 2023). Kiik et al. (2017) highlighted the lack of family focussed nursing education, with the current focus on the Biomedical Model identified as one of the major factors hindering nurses and other health care professionals in collaborating with families. Despite these obstacles, family nurse researchers across the globe continue to encourage nursing undergraduate programmes to include family nursing theory, family nursing constructs and family nursing interventions to enhance knowledge and skills training in FNP, thus enabling the students to meet families’ needs and deliver optimal family engagement (Eggenberger & Regan 2010; Coyne 2015; Meiers et al. 2018; Skene et al. 2019; Chironda et al. 2023).

Although studies of FNPs are gaining momentum in global literature, the available evidence has largely targeted the qualified nursing professionals (Imanipour & Kiwanuwa 2020; Misto 2014, 2018; Naef et al. 2020), with limited studies exploring this phenomenon in undergraduate student nurses. Hence the aim of the study was to explore the undergraduate nurse perceptions of FNPs in a selected institution in KwaZulu–Natal (KZN).

## Research methods and design

### Study design and setting

A descriptive survey design was used to explore the undergraduate student nurses’ perceptions of their FNPs at selected institution in KZN. Use of this design enabled the participants at the centre of the research problem to provide quantitative and qualitative data, which is relevant and accurate as indicated by Siedlecki (2020). The study was conducted at a selected university, which offers health science courses including the nursing programme. The nursing discipline that started in 1956 at diploma level is housed under the college of health sciences within the School of Nursing and Public Health. Currently, the discipline has grown and is now offering undergraduate and postgraduate nursing programmes. Additionally, the discipline is a World Health Organization Collaborating Centre for Nursing and Midwifery Development in the African Region. The institution had been offering the old undergraduate nursing curriculum (R425) up to 2019 and the new curriculum (R171) currently from 2020. Focussing on this setting increased the likelihood of getting study participants who are being trained at a level required by the researchers.

### Population, sample and sampling

Research respondents were an all-inclusive sample of 2nd year (88) and 3rd year (66) (*N* = 154) students currently registered for a baccalaureate nursing programme. First- and 4th-year undergraduate nursing students were excluded because of insufficient clinical exposure and insufficient time, respectively. The final sample size used was 77. Purposive sampling strategy was used as the researchers wanted to gather information from only participants in 2nd and 3rd level as they have already met families during their exposure to clinical areas. According to Creswell (2014), purposive sampling involves identifying and selecting groups of individuals that are experienced with a phenomenon of interest.

### Data collection instrument

The Family Nursing Practice Scale (FNPS) (Simpson & Tarrant 2006) was developed using the frameworks of Family Systems Nursing, the Calgary Family Assessment Model and the Calgary Family Interventional Model (Simpson & Tarrant 2006) and has been widely used in nursing studies around the world (Hsiao & Tsai 2015; Imanipour & Kiwanuwa 2020; Misto 2018; Naef et al. 2020). Permission to use the tool was obtained from the authors (Simpson & Tarrant 2006). This tool has two subscales namely practice appraisal (PA) (5 items) and NFR (5 items) and a demographic section (3 items). The PA subscale measures nurses’ attitudes on working with families and critical appraisal of their own FNP (knowledge, skills, confidence and satisfaction). The NFR subscale measures perceptions of interaction and reciprocity in the NFR that is, planning care, promoting family participation and recognising biases and reciprocity in the therapeutic relationship (Simpson & Tarrant 2006). The responses of the FNPS items are rated on a 5-point Likert-type scale ranging from 1 (high) to 5 (low). The scale is reverse coded; thus a lower score per subscale (inverse score of < 2.2) indicates higher critical PA of FNP and higher perceptions of interaction and reciprocity in the NFR, while a higher score per subscale (inverse score of greater than 2.9) indicates lower critical PA and lower perceptions of interaction and reciprocity in the NFR (Naef et al. 2020; Simpson & Tarrant 2006).

The questionnaire also includes three open-ended questions regarding the challenges and advantages of involving the family in assessment and care planning, as well as asking about how the student nurses currently included families in their nursing practice.

### Validity and reliability

Family nursing practice scale was previously evaluated for face and content validity using content review experts and factor analysis for construct validity. The items within the instrument were made from aspects of literature review, needs assessment with nurses, a family systems approach and a competency framework including review using focus group (Naef et al. 2020; Simpson & Tarrant 2006). The item loadings factor for all the items ranged from 0.801 to 0.506, which accounted 41.9% and 14.5% of the variance for PA and NFR practice subscale, respectively (Simpson & Tarrant 2006).

A pretesting was conducted electronically with three 2nd and 3rd year students prior to data collection to ensure that the tool was clear and easy to understand. The students involved in pretesting were not included in the final sample size. No alterations were made to the tool and the results of were not included in the study. The FNPS previously tested Cronbach’s alpha reliability coefficients were 0.88 and 0.73 for the PA and nurse family relationships, respectively, with 0.86 for the scale overall (Simpson & Tarrant 2006) thus indicating high internal consistency. Test-retest reliability ranged from 0.62 to 0.93 on the individual items revealing evidence of the reliability and validity of the FNPS (Simpson & Tarrant 2006). Other studies reported an overall Cronbach’s alpha reliability coefficients of 0.80 (Naef et al. 2020), ≥ 0.85 (Hsiao & Tsai 2015) and 0.87 (Svavarsdottir et al. 2018). For the current study, Cronbach’s alpha reliability coefficient was 0.81, meaning the instrument was a very good measure of the concepts under study. According to Taber (2018, Cronbach alpha values of 0.7 or higher indicate acceptable internal consistency.

### Data collection process

Data collection took place from October to December 2021. Because of COVID-19 research regulations, the questionnaire was administered to respondents electronically using Survey Monkey (SurveyMonkey.com, LLC). The first part of the online questionnaire asked for consent, and upon agreeing, the respondents were automatically allowed to complete the rest of the questionnaire. All the responses were linked to the email address of the researcher (G.C.).

### Data analysis

With the help of a statistician, quantitative data were cleaned, coded, and entered into IBM Statistical Package for the Social Sciences (SPSS) version 26 in preparation for analysis (Pallant, 2020). Descriptive statistics were used to describe the demographic data and the family practices among the respondents. The student T test was used to identify any significance differences in responses of items for PA and NFR subscales. Qualitative content analysis (Erlingsson & Brysiewicz 2017) was used to analyse the three open-ended questions.

### Ethical considerations

Permission to conduct the study was obtained from the ethics committee of the university of KwaZulu–Natal, (Ethical clearance number: HSSREC/00002104/2020).

## Results

### Demographic characteristics

Out of 154 undergraduate nursing students, the response rate was 50% (*N* = 77). Respondents were predominantly female, in the age group of 18 years to 23 years and over half were 2nd year undergraduate nursing students (Table 1).

**TABLE 1:** Demographic characteristics of the study population (*N* = 77).

Variable	Frequency	%
**Age in years**		
18–23	73	55.8
24–30	4	41.5
**Sex**		
Male	10	13.0
Female	67	87.0
**Year of study**		
Year 2	43	55.8
Year 3	34	44.2

*Source:* Adapted from Simpson, P. & Tarrant, M., 2006, ‘Development of the family nursing practice scale’, *Journal of Family Nursing* 12(4), 413–425. https://doi.org/10.1177%2F1074840706290806

### Undergraduate student nurses’ perceptions of family nursing practices

Undergraduate student nurses rated their overall FNP as low (M = 3.43, s.d. = 0.99). Females (M = 3.01) and 24 years to 30 years age group (M = 2.88) exhibited better FNP compared to males (M = 3.21) and 18 to 23 years (3.38), respectively. Further analysis revealed higher FNP (2.97) was recorded for 3rd year nursing students as compared to 2nd year (3.90) nursing students with significance differences in the means (*p* < 0.0001) (Table 2).

**TABLE 2:** Family nursing practices among the 2nd and 3rd year nursing students (*N* = 77).

Descriptor	Total (*N* = 77)		2nd (*n* = 43)		3rd (*n* = 34)		*t*-test	Sig. (2-tailed)
	Mean	s.d.	Mean	s.d.	Mean	s.d.		
**Practice appraisal (PA)**								
PA1	3.25	0.85	3.47	1.08	3.02	1.17	−3.901	0.000*
My confidence level in working with families is								
PA2	3.72	0.91	4.15	1.02	3.28	1.32	−3.168	0.002*
My level of satisfaction with family nursing is								
PA3	3.44	0.77	3.91	0.99	2.96	1.09	−4.178	0.000*
My knowledge level of family system nursing is								
PA4	3.38	0.87	3.94	0.81	2.81	0.96	−2.824	0.006*
My skill in working with the family system is								
PA5	3.38	0.82	3.68	0.98	3.07	1.01	−2.657	0.010*
I feel comfortable in initiating family involvement in nursing care planning								
Subscale mean score	3.43	0.84	3.83	0.96	3.02	0.99	−2.768	0.003
**Nurse Family Relationship (NFR)**								
NFR1	3.59	0.97	4.03	0.83	3.14	1.13	−3.849	0.000*
I plan nursing interventions in consultation with the patient and family								
NFR2	3.48	0.92	4.05	1.22	2.91	0.03	−4.284	0.000*
Families always approach me about their ill relatives								
NFR3	3.15	0.76	3.82	1.03	2.47	1.09	−5.539	0.000*
I promote family and patient participation, choice and control in meeting health care needs								
NFR4	3.66	0.95	4.06	0.98	3.26	1.07	−3.386	0.001*
My involvement with family is mostly rewarding								
NFR5	3.33	0.81	3.82)	1.03	2.84	0.89	−4.487	0.000*
I avoid interferences of my own biases when collecting, interpreting and communicating data about patients and families								
Subscale mean score	3.44	0.88	3.96	1.02	2.92	-	−4.234	0.000*

**Overall total mean score**	**3.43**	**0.86**	**3.90**	**0.99**	**2.97**	**-**	**−4.131**	**0.000** *

*Source:* Adapted from Simpson, P. & Tarrant, M., 2006, ‘Development of the family nursing practice scale’, *Journal of Family Nursing* 12(4), 413–425. https://doi.org/10.1177%2F1074840706290806

s.d., standard deviation.

* , Significance level set at 0.05.

#### Practice appraisal

Student nurses reported their critical PA subscale with a mean and s.d. of 3.43 and 0.58, respectively. Despite this, 3rd year student nurses critically appraised their FNP better than 2nd year student nurses. Further analysis of the FNP-PA for 2nd year students showed that the items ‘My level of satisfaction with family nursing is’ (3.72), ‘My skill in working with the family system is’ (3.38) and ‘My knowledge level of family system nursing is’ (3.44) all had the highest mean scores as compared to 3rd year students. Thus, these results translate to a low critical PA in terms of satisfaction, skill and knowledge for the 2nd year year nursing students compared to the 3rd year nursing students (Table 2).

#### Nurse-family relationship

The reported mean and s.d. for the FNP-NFR subscale was 3.43 and 0.47, respectively. As expected, 3rd year student nurses reported their interaction and reciprocity in the NFR higher than 2nd year students. The FNP-NFR on item ‘I plan nursing interventions in consultation with the patient and family’ (4.03), families always approach me about their ill relatives’ (4.05) and ‘My involvement with family is mostly rewarding’ (4.06) had higher means for second than for 3rd year nursing students. Therefore, lower perceptions of interaction and reciprocity in the NFR in terms of planning care and promoting family participation were reported by the 2nd year nursing students (Table 2).

#### Student nurses’ qualitative perceptions of family nursing practice

The categories of identified challenges included family conflict, maintaining confidentiality, lacks skills, not prepared and/or ill prepared and absent family. The advantages were obtaining of detailed information, ability to plan individualised care and enhanced student NFR. Current FNPs were conducted for family assessments including giving family health education (Table 3).

**TABLE 3:** Responses to the open-ended questions.

Category	Number of Responses	Example of a response
**Challenges of family involvement**		
Family conflict	30	‘Not everyone will always agree to one thing. Some members know much more than others, so they conflict and sometimes fight when giving information for the patient.’ (3rd year)
Maintaining confidentiality	31	‘Some families protect their family secrets, so they don’t provide full information that is helpful when dealing with patient.’ (3rd year)
Not prepared and/or ill prepared	33	‘We are not taught anything on families, so it becomes difficult to communicate with families.’ (2nd year)
Absent family	25	‘Family members are not always available when we need their help in dividing which intervention are needed to improve condition of the patients.’ (3rd year)
**Advantages of family involvement**		
Detailed information	38	‘Families gives comprehensive background history of the patient, and this helps us nurses to understand the healthcare needs including the general family structure and composition.’ (2nd year).
Ability to plan individualised care	35	‘You are able to get the ideas from the family, plan the interventions and modify the care plan in way that will suit the patient and family.’ (3rd year)
Enhanced student nurse-family relationship.	33	‘Involving families helps student nurses to build trust and long-lasting mutual relationships with the patient and respective families. The nurses get to know families personally and understand how they perceive the illness of a family member.’ (2nd year).
**Examples of current family nursing practices**		
Family health education	25	‘A patient was being discharged from the hospital. He had to be given information on the next follow up consultations and the medication he was taking home. As I was accompanying the patient out, I had to hand the information over to the patient’s wife.’ (3rd year)
Family assessment	34	‘I conducted a family study in which I chose a family in the community I’m allocated in to investigate or find out about the family’s situation, lifestyle or environment and roles of each family member. This family study mainly focused on the child’s [*3 year old boy*] development, health, etc. I visited this family, interviewed them and collected the data and information from them including info about the development and health of the 3 year old son to conduct a family study based on this family.’ (2nd year)

*Source:* Adapted from Simpson, P. & Tarrant, M., 2006, ‘Development of the family nursing practice scale’, *Journal of Family Nursing* 12(4), 413–425. https://doi.org/10.1177%2F1074840706290806

## Discussion

The undergraduate student nurses’ critical appraisal of their FNP, perceptions of interaction and reciprocity in the NFR including the challenges, advantages and examples of how they worked with families during their nursing practicum were explored in this descriptive study.

The overall mean total score indicated low FNPs for undergraduate nursing students in keeping with the findings of a study done by Lee, Leung and Mak (2012) in Hong Kong. While there are limited studies on student nurses’ assessment of their FNP, the available literature has explored qualified nurses’ perceptions across the globe (Imanipour & Kiwanuka 2020; Misto 2014; Naef et al. 2020, 2021). The studies done by Misto (2014) and Naef et al. (2020) revealed high FNPs while some indicated moderate practices (Imanipour & Kiwanuka 2020; Naef et al. 2021). This is not surprising as family nursing content in undergraduate curricula is still being delivered embedded within other subject areas like community health nursing in many nursing schools (Honda 2021).

Similarly, the South African Nursing Council (SANC) undergraduate curriculum, both the old (R425 registered nurse) and new (R171 Professional Nurse and Midwife), includes a community nursing qualification with an embedded FNP (SANC 2021). The same applies to the postgraduate programmes in Primary Health Care (PHC) and community health nursing. Hence, both 2nd and 3rd year undergraduate nursing students embraced their current FNPs through family assessments including giving family health education during community nursing practice. However, this seems to be inadequate as the students lacks more in-depth knowledge and the skill of FNP.

A greater number of female nursing students’ representation relate to nursing being a feminine gendered profession and this deters most men from choosing it as a lifelong career (Van der Cingel & Brouwer 2021). While the proportion of undergraduate student male nurses in the sample was low, they still exhibited a significant low family practice than their female counterparts. These findings might be because of differences in culture and communication styles in males and females (Cranley et al. 2022). In the South African cultural context, females from the young age are taught to perform motherly duties (Bassey & Bubu 2019), this might explain why undergraduate female nursing students in the current study had better FNPs. Similarly, males and females have different communication skills because of differences in one’s socialisation (Cranley et al. 2022). According to Arreciado Marañón, Rodríguez-Martín and Galbany-Estragués (2019), male nurses limit their emotional involvement and take control over the boundaries of relationships with families as compared to their female colleagues. Contrarily, a study by Hoplock et al. (2019) revealed no significant association between gender and nurses’ attitudes towards the importance of family involvement in nursing care.

Findings of the study confirm 3rd year nursing students to have rated their FNPs better than 2nd year students. Although there are no studies to affirm or contrast the findings in the current study, similar trends are shown in qualified nurses, where nurses with graduate education exhibited higher FNPs than those with bachelors (Svavarsdottir et al. 2018). Similarly, on FNP-PA subscale (Simpson & Tarrant 2006), 2nd year student nurses expressed lower critical appraisal of family nursing than 3rd year students thus indicating less confidence especially in skills, knowledge of family systems and satisfaction in working with families. Eggenberger, Krumwiede and Young (2015) reported lack of confidence in nurses’ ability to meet the needs of families. Moreover, student nurses avoid families, and they always feel anxious when meeting with them (Shajan & Snell 2019) probably because of their limited knowledge and skills. This is expected as there is little training available on nursing of families (Wittenberg et al. 2020) and most nursing education has not fully embraced the incorporation of family nursing into their undergraduate curriculums (Chironda et al. 2023). Undergraduate nursing programmes need to teach family nursing to evoke ‘thinking family’ in students’ nursing practice (Fast Braun, Hyndman & Foster 2010).

The need for more education about family nursing and involving families in the care of patient is apparent as student indicated that they are inadequate and ill prepared in their current training, thus concurring with the findings from Kaakinen et al. (2018) and Gutiérrez-Alemán et al. (2021). Sadly, students also felt undermined with family members dominating and purporting to know what is best for the patient. Therefore, it is the duty of the nurse educators to create and implement curriculum centred on FNP (Meiers et al. 2018). It has been suggested that using an educational intervention on family members in health settings have helped nurses to ‘think family’ with improved competence in family nursing conversations (Broekema et al. 2018).

Similarly, low perceptions of interaction and reciprocity in the NFR were identified on the FNP-NFR subscale especially in the 2nd year students, thus contrasting the findings of Lee et al. (2012). While Kydonaki et al. (2019) emphasised the importance of frequent communication with families as the basis for establishment of rapport and trust, undergraduate nursing students confirmed refusal of families to open up enough to provide any information as they were not confident in their capabilities. Consequently, families could not avail correct or omit essential information because they cannot trust the nursing staff. While nurses have an ethical and legal obligation to engage families in nursing care (Shajan & Snell 2019), sometimes conflicting views either within the family or with the student nurses occur because of family feuds or differences in perspectives as revealed in the study, thus affirming the finding of Misto (2014, 2018). In support of this, an integrative review by Rainer, Schneider and Lorenz (2018) reveals inappropriate behaviour or hostility towards nursing staff by families, thus resulting in conflict between the parties.

Acquiring detailed information about the patient was indicated by the student nurses, thus showing families as valuable especially in vulnerable conditions such as hospitalisation (Barreto et al. 2022; Ruiz et al. 2022). Ability to plan individualised care and enhanced student NFR is another benefit of involving family thus confirming the findings of Shajan and Snell (2019) and Misto (2018). Coats et al. (2018) appreciated the importance of family presence in allowing nurses to get to know families better and their preferences for care. Further family assessments done by student nurses ranged from engagements, identifying healthcare needs, taking medical and surgical history and involving families to discuss patient condition including discharge planning, thus agreeing to the findings of Misto (2018). Moreover, a 14 2-h classes of teaching students to work with families enhanced the critical appraisal of FNP and reflections on the NFR in undergraduate nursing students in Hong Kong (Lee et al. 2012). Nursing curriculums with family nursing content have shown to improve students’ knowledge of family and instil a passion for family care in undergraduate programmes (Meiers et al. 2018).

### Study limitations

The study was conducted in one nursing institution; therefore the findings cannot be generalised to other settings. Participants were comprehending and giving information depending on how they understood it. Moreover, the level of knowledge experience in work integrated learning between the 2nd and 3rd year nursing students was different; hence the exclusion of 4th year students could be a limitation in this study. Because of COVID-19 pandemic, only online methods of data collection were used.

### Recommendations

While 2nd year undergraduate student nurses embraced getting full detailed history and improved family nurse relationships through family involvement, they still require the detailed theoretical component of family nursing course prior to community attachment to acquire the relevant knowledge. Similarly, nurse educators are also recommended to create and integrate family nursing concepts into theory during content delivery. For the 3rd year undergraduate nursing students, strengthening of FNP through use of innovative teaching strategies such as simulation is recommended to improve the skill of dealing with family conflicts and absent family members. This will enhance further application of these skills in Work Integrated Learning (WIL) where nursing care plans are formulated, and nursing interventions are implemented to patients.

## Conclusion

Compared to 3rd year students, a lower critical appraisal of FNP and lower perceptions of interaction and reciprocity in the NFR were identified among 2nd year nursing students. Based on the *p* value of 0.001, there is a very low probability of 0.1% that these results are because of random chance, meaning the results are true. Obtaining detailed information, ability to plan individualised care and enhanced student NFR were advantages, while family conflict, maintaining confidentiality, ill prepared and absent family were challenges experienced in FNP. Although the SANC undergraduate and postgraduate programmes in PHC and community embraces family nursing, there is also need for an inclusive curriculum that promotes and advocates for family nursing within the undergraduate programme.

## References

[CIT0001] Abrams, M.A., Gail Nielsen, B.S.H.C.A., Karen Frush, B.S.N. & Balik, B., 2013, ‘Ensuring patient involvement and family engagement’, *The Essential Guide for Patient Safety Officers*, p. 91, viewed n.d., from https://www.dca.org.sa/downloads/dca/quality_gate/04_E-Library/Healthcare%20Quality/Essential%20guide%20for%20patient%20safety%20officers%20,%202nd%20ed..pdf#page=104.

[CIT0002] Aronson, P.L., Yau, J., Helfaer, M.A. & Morrison, W., 2009, ‘Impact of family presence during pediatric intensive care unit rounds on the family and medical team’, *Pediatrics* 124(4), 1119–1125. 10.1542/peds.2009-0369 19736262

[CIT0003] Arreciado Marañón, A., Rodríguez-Martín, D. & Galbany-Estragués, P., 2019, ‘Male nurses’ views of gender in the nurse–family relationship in paediatric care’, *International Nursing Review* 66(4), 563–570. 10.1111/inr.12541 31373386

[CIT0004] Barreto, M.D.S., Marquete, V.F., Camparoto, C.W., García-Vivar, C., Barbieri-Figueiredo, M.D.C. & Marcon, S.S., 2022, ‘Factors associated with nurses’ positive attitudes towards families’ involvement in nursing care: A scoping review’, *Journal of Clinical Nursing* 31(23–24), 3338–3349. 10.1111/jocn.16226 35083808 PMC9786255

[CIT0005] Bassey, S.A. & Bubu, N.G., 2019, ‘Gender inequality in Africa: A re-examination of cultural values’, *Cogito* 11(3), 21–36, viewed 20 August 2023, from https://www.ceeol.com/search/article-detail?id=884671.

[CIT0006] Broekema, S., Luttik, M.L.A., Steggerda, G.E., Paans, W. & Roodbol, P.F., 2018, ‘Measuring change in nurses’ perceptions about family nursing competency following a 6-day educational intervention’, *Journal of Family Nursing* 24(4), 508–537. 10.1177/1074840718812145 30453803

[CIT0007] Broekema, S., Paans, W., Roodbol, P.F. & Luttik, M.L.A., 2021, ‘Effects of family nursing conversations on families in home health care: A controlled before-and-after study’, *Journal of Advanced Nursing* 77(1), 231–243. 10.1177/1074840718812145 33068016

[CIT0008] Chironda, G. & Brysiewicz, P., 2021, *Nursing is much more than just focusing on the patient. Towards Research Leadership*, Jive Media Africa, viewed 08 December 2022, from http://hdl.handle.net/1451549773908033552.

[CIT0009] Chironda, G., Jarvis, M.A. & Brysiewicz, P., 2023, ‘Family-focused nursing research in WHO Afro-Region Member states: A scoping review’, *Journal of Family Nursing* 29(2), 136–154. 10.1177/10748407221132018 36433834 PMC10160405

[CIT0010] Coats, H., Bourget, E., Starks, H., Lindhorst, T., Saiki-Craighill, S., Curtis, J.R. et al., 2018, ‘Nurses’ reflections on benefits and challenges of implementing family-centred care in paediatric intensive care units’, *American Journal of Critical Care* 27(1), 52–58. 10.4037/ajcc2018353 29292276 PMC5959722

[CIT0011] Coyne, I., 2015, ‘Families and health-care professionals’ perspectives and expectations of family-centered care: hidden expectations and unclear roles’, *Health Expectations* 18(5), 796–808. 10.1186/s12904-015-0032 23800327 PMC5060842

[CIT0012] Cranley, L.A., Lam, S.C., Brennenstuhl, S., Kabir, Z.N., Boström, A.M., Leung, A.Y.M. et al., 2022, ‘Nurses’ attitudes toward the importance of families in nursing care: A multinational comparative study’, *Journal of Family Nursing* 28(1), 69–82. 10.1177/10748407211042338 34493109 PMC8814953

[CIT0013] Creswell, J.W., 2014, *A concise introduction to mixed methods research*, SAGE publications. https://books.google.com/books?hl=en&lr=&id=51UXBAAAQBAJ&oi=fnd&pg=PR1&dq=creswell,+2014&ots=6bCnP7WqLB&sig=cCrXkS34eB0Ilv7hp7-z8Wp_KzE

[CIT0014] DePoy, E. & Gitlin, L.N., 2016, *Research ethics in introduction to research*, 5th edn., viewed 05 December 2022, from https://www.sciencedirect.com/science/article/pii/B9780323261715000033.

[CIT0015] Eggenberger, S.K. & Regan, M., 2010, ‘Expanding simulation to teach family nursing’, *Journal of Nursing Education* 49(10), 550–558. 10.3928/01484834-20100630-01 20672781

[CIT0016] Eggenberger, S.K. & Sanders, M., 2016, ‘A family nursing educational intervention supports nurses and families in an adult intensive care unit’, *Australian Critical Care* 29(4), 217–223. 10.1016/j.aucc.2016.09.002 27688123

[CIT0017] Eggenberger, S.K., Krumwiede, N.K. & Young, P.K., 2015, ‘Using simulation pedagogy in the formation of family-focused generalist nurses’, *Journal of Nursing Education* 54(10), 588–593. 10.3928/01484834-20150916-08 26431520

[CIT0018] Erlingsson, C. & Brysiewicz, P., 2017, ‘A hands-on guide to doing content analysis’, *African Journal of Emergency Medicine* 7(3), 93–99. 10.1016/j.afjem.2017.08.001 30456117 PMC6234169

[CIT0019] Fast Braun, V., Hyndman, K. & Foster, C., 2010, ‘Family nursing for undergraduate nursing students: The Brandon University family case model approach’, *Journal of Family Nursing* 16(2), 161–176. https://doi.org/10.1177%2F1074840710366565 20406999 10.1177/1074840710366565

[CIT0020] Fitzgerald, M. & Ward, J., 2019, ‘Using standardized actors to promote family-centered care’, *Journal of Pediatric Nursing* 45, 20–25. 10.1016/j.pedn.2018.12.002 30594888

[CIT0021] Goodridge, D., Henry, C., Watson, E., McDonald, M., New, L., Harrison, E.L. et al., 2018, ‘Structured approaches to promote patient and family engagement in treatment in acute care hospital settings: Protocol for a systematic scoping review’, *Systematic Reviews* 7(1), 1–7. 10.1186/s13643-018-0694-9 29482622 PMC5827976

[CIT0022] Gutiérrez-Alemán, T., Esandi, N., Pardavila-Belio, M.I., Pueyo-Garrigues, M., Canga-Armayor, N., Alfaro-Diaz, C. et al., 2021, ‘Effectiveness of educational programs for clinical competence in family nursing: A systematic review’, *Journal of Family Nursing* 27(4), 255–274. 10.1177/10748407211038683 34420376

[CIT0023] Hagedoorn, E.I., Paans, W., Jaarsma, T., Keers, J.C., Van der Schans, C.P. & Luttik, M.L.A., 2021, ‘The importance of families in nursing care: Attitudes of nurses in the Netherlands’, *Scandinavian Journal of Caring Sciences* 35(4), 1207–1215. 10.1111/scs.12939 33270268

[CIT0024] Honda, J., 2021, ‘Educação Para Melhorar A Competência Da Enfermagem Familiar/Education for Improving Family Nursing Competency’, *Revista Paranaense De Enfermagem (Repenf)* 4(1), 39–41. https://www.researchgate.net/profile/Junko-Honda/publication/348364354_EDUCATION_FOR_IMPROVING_FAMILY_NURSING_COMPETENCY/links/5ffaa7e1a6fdccdcb8436fbf/EDUCATION-FOR-IMPROVING-FAMILY-NURSING-COMPETENCY.pdf.

[CIT0025] Hoplock, L., Lobchuk, M., Dryburgh, L., Shead, N. & Ahmed, R., 2019, ‘Canadian hospital and home visiting nurses’ attitudes toward families in transitional care: A descriptive comparative study’, *Journal of Family Nursing* 25(3), 370–394. 10.1177/1074840719863499 31328621

[CIT0026] Hsiao, C.Y. & Tsai, Y.F., 2015, ‘Factors associated with the perception of family nursing practice among mental health nurses in Taiwan’, *Journal of Family Nursing* 21(4), 508–528. 10.1177/1074840715606543 26410853

[CIT0027] Imanipour, M. & Kiwanuka, F., 2020, ‘Family nursing practice and family importance in care–Attitudes of nurses working in intensive care units’, *International Journal of Africa Nursing Sciences* 13, 100265. 10.1016/j.ijans.2020.100265

[CIT0028] Kaakinen, J.R., Coehlo, D.P., Steele, R. & Robinson, M., 2018, *Family health care nursing: Theory, practice, and research*, FA Davis, viewed n.d., from https://books.google.com/books?hl=en&lr=&id=wNFJDwAAQBAJ&oi=fnd&pg=PR1&dq=Kaakinen,+J.R.,+Coehlo,+D.P.,+Steele,+R.+and+Robinson,+M.,+2018.+Family+health+care+nursing:+Theory,+practice,+and+research.+FA+Davis&ots=29lMqm5no1&sig=5p7T_e-x0W0kksRBCaRd5NzivRI.

[CIT0029] Kiik, S.M., Mahathir, M. & Prasetyono, J.D., 2017, ‘Classroom simulation method increases nursing student family-focused care competencies in family nursing learning practice’, *Indonesian Nursing Journal of Education And Clinic (INJEC)*, 2(1), 71–76. 10.24990/injec.v2i1.129

[CIT0030] Kydonaki, K., Kean, S. & Tocher, J., 2020, ‘Family Involvement in intensive care: A qualitative exploration of critically ill patients, their families and critical care nurses (INpuT study)’, *Journal of Clinical Nursing* 29(7-8), 1115–1128. 10.1111/jocn.15175 31889366

[CIT0031] Lee, A.C., Leung, S.S. & Mak, Y.W., 2012, ‘The application of family-nursing assessment skills: From classroom to hospital ward among final-year nursing undergraduates in Hong Kong’, *Nurse Education Today* 32(1), 78–84. 10.1016/j.nedt.2011.01.013 21345549

[CIT0032] Meiers, S.J., Eggenberger, S.K. & Krumwiede, N., 2018, ‘Development and implementation of a family-focused undergraduate nursing curriculum: Minnesota State University, Mankato’, *Journal of Family Nursing* 24(3), 307–344. 10.1177/1074840718787274 30101655

[CIT0033] Misto, K., 2014, *The relationship between families’ perceptions and nurses’ perceptions of family nursing practice*, University of Rhode Island, viewed 22 July 2023, from https://search.proquest.com/openview/3542d8802bcfd51dff9d46629ea7e4ab/1?pq-origsite=gscholar&cbl=18750.

[CIT0034] Misto, K., 2018, ‘Nurse perceptions of family nursing during acute hospitalizations of older adult patients’, *Applied Nursing Research* 41, 80–85. 10.1016/j.apnr.2018.04.009 29853220

[CIT0035] Naef, R., Ernst, J., Müeller, M. & Schmid-Mohler, G., 2021, ‘Translation and psychometric validation of the German version of the Family Nursing Practice Scale (FNPS)’, *Journal of Family Nursing* 27(1), 34–42. 10.1016/j.aucc.2016.09.002 33183149

[CIT0036] Pallant, J., 2020, *SPSS survival manual: A step by step guide to data analysis using IBM SPSS*, McGraw-hill education, viewed n.d., from https://books.google.com/books?hl=en&lr=&id=CxUsEAAAQBAJ&oi=fnd&pg=PP1&dq=IBM+SPSS+version+27&ots=n40yzIJ67S&sig=PjtkiDAF_Ku2yZ2NY1Idg4nQx2A.

[CIT0037] Rainer, J., Schneider, J.K. & Lorenz, R.A., 2018, ‘Ethical dilemmas in nursing: An integrative review’, *Journal of Clinical Nursing* 27(19–20), 3446–3461. 10.1111/jocn.14542 29791762

[CIT0038] Ruiz, A.G.B., Marcon, S.S., Haddad, M.D.C.F.L., Kalinke, L.P., Teston, E.F., Schwartz, E. et al., 2022, ‘Construct validity and reliability of the scale Families’ Importance in Nursing Care-Nurses’ attitudes’, *Acta Paulista de Enfermagem* 35, eAPE039019234. 10.37689/acta-ape/2022AO01924

[CIT0039] SANC, 2021, *South African Nursing Council Annual report 2021–2022*, viewed 05 October 2023, from https://pmg.org.za/files/SANC-Annual-Report-2021-2022.pdf.

[CIT0040] Shajan, Z. & Snell, D., 2019, *Wright & Leahey’s nurses and families: A guide to family assessment and intervention*, FA Davis, viewed n.d., from https://books.google.com/books?hl=en&lr=&id=lsOHDwAAQBAJ&oi=fnd&pg=PR1&dq=Shajan,+Z.+and+Snell,+D.,+2019.+Wright+%26+Leahey%27s+nurses+and+families:+A+guide+to+family+assessment+and+intervention.+FA+Davis.+&ots=FiszXdMdan&sig=6WEg1Ta5aOyvyI6YwGJS_6fcyfo.

[CIT0041] Siedlecki, S.L., 2020, ‘Understanding descriptive research designs and methods’, *Clinical Nurse Specialist* 34(1), 8–12. 10.1097/NUR.0000000000000493 31789957

[CIT0042] Simpson, P. & Tarrant, M., 2006, ‘Development of the family nursing practice scale’, *Journal of Family Nursing* 12(4), 413–425. https://doi.org/10.1177%2F1074840706290806 17099118 10.1177/1074840706290806

[CIT0043] Skene, C., Gerrish, K., Price, F., Pilling, E., Bayliss, P. & Gillespie, S., 2019, ‘Developing family-centred care in a neonatal intensive care unit: An action research study’, *Intensive and Critical Care Nursing* 50, 54–62. 10.1016/j.iccn.2018.05.006 29937077

[CIT0044] South African government, 2013, *Protection of Personal Information Act 4 of 2013*, viewed 07 December 2022, from https://www.gov.za/sites/default/files/gcis_document/201409/3706726-11act4of2013popi.pdf.

[CIT0045] Svavarsdottir, E.K., Sigurdardottir, A.O., Konradsdottir, E. & Tryggvadottir, G.B., 2018, ‘The impact of nursing education and job characteristics on nurse’s perceptions of their family nursing practice skills’, *Scandinavian Journal of Caring Sciences* 32(4), 1297–1307. 10.1111/scs.12573 29691883

[CIT0046] Taber, K.S., 2018, ‘The use of Cronbach’s alpha when developing and reporting research instruments in science education’, *Research in Science Education* 48, 1273–1296. 10.1007/s11165-016-9602-2

[CIT0047] Van der Cingel, M. & Brouwer, J., 2021, ‘What makes a nurse today? A debate on the nursing professional identity and its need for change’, *Nursing Philosophy* 22(2), e12343. 10.1111/nup.12343 33450124

[CIT0048] Wiles, R., Crow, G., Heath, S. & Charles, V., 2008, *Anonymity and confidentiality*, e ESRC Research Methods Festival, University of Oxford, viewed 02 October 2023, from http://www.sociology.soton.ac.uk/proj/informed_consent/index.htm.

[CIT0049] Wittenberg, E., Goldsmith, J.V., Williams, Y.E. & Lee, A., 2020, ‘Caring for family caregivers: A pilot test of an online COMFORT™ SM communication training module for undergraduate nursing students’, *Journal of Cancer Education* 35(1), 138–143. 10.1007/s13187-018-1452-3 30467775 PMC6533166

